# Identification of a Lactic Acid Bacteria to Degrade Biogenic Amines in Chinese Rice Wine and Its Enzymatic Mechanism

**DOI:** 10.3390/foods8080312

**Published:** 2019-08-02

**Authors:** Tianjiao Niu, Xing Li, Yongjie Guo, Ying Ma

**Affiliations:** 1 School of Chemistry and Chemical Engineering, Harbin Institute of Technology, Harbin 150090, China; 2 Mengniu Hi-tech Dairy (Beijing) Co., Ltd., Beijing 101107, China

**Keywords:** biogenic amines, *L. plantarum*, amines oxidase, Chinese rice wine, industrial fermentation

## Abstract

A *L. plantarum*, CAU 3823, which can degrade 40% of biogenic amines (BAs) content in Chinese rice wine (CRW) at the end of post-fermentation, was selected and characterized in this work. It would be an optimal choice to add 10^6^ cfu/mL of selected strain into the fermentation broth to decrease the BAs while keeping the character and quality of CRW. Nine amine oxidases were identified from the strain and separated using Sephadex column followed by LC-MS/MS analysis. The purified amine oxidase mixture showed a high monoamine oxidase activity of 19.8 U/mg, and more than 40% of BAs could be degraded. The biochemical characters of the amine oxidases were also studied. This work seeks to provide a better solution to degrade BAs in CRW prior to keeping the character and quality of CRW and a better understanding of the degradability of the strain to the BAs.

## 1. Introduction

Biogenic amines (BAs) are low molecular weight organic compounds that have been identified as toxicological agents in various foods, such as fishery products, dairy, meat, wine, and so on [1,2]. The ingestion of foods containing relatively high concentrations of BAs could lead to several health hazards, such as headaches, hypotension, respiratory distress, heart palpitations and digestive problems, particularly when alcohol is present [3,4]. Histamine, which is well-known because of its implication in many food poisoning cases, has a potent vasodilatory action that could cause important drops in blood pressure [5]. Tyramine, as one of the vasoconstrictor amines, can provoke a release of noradrenaline resulting in an increase of arterial pressure [5]. Even though there are no accurate regulations for BAs, several countries including France, Germany and Australia have set regulations and limits for histamine and many wine importers in the EU require a BA analysis [4,6]. The presence of BAs is considered a marker of poor wine quality and bad winemaking practices [4,7].

BAs are synthesized in fermented food by decarboxylation of corresponding amino acids by microorganisms [1]. According to the previous studies, BAs could be formed by lactic acid bacteria in wine [8,9], Chinese rice wine [10] and Korean rice wine [1]. As a traditional alcoholic beverage, Chinese rice wine (CRW), which has been popular in China for thousands of years [11], has high nutritional values, and thus, it has been used as an ingredient in traditional Chinese medicine [12]. Since the brewing process of CRW is the typical open semisolid-state fermentation, lots of microorganisms (molds, yeast, bacteria) are brought in the glutinous rice with the addition of Chinese koji [3,13], and the system is favorable to BAs generation combining with the high amount of free amino acids [2]. The abundant bacteria in CRW, mainly originating from Chinese koji, the surroundings and the surfaces of the equipment, could be one of the main reasons for the formation of BAs [10].

Histamine, tyramine, putrescine, cadaverine and phenylethylamine are the most representative BAs detected in the wine [6]. Histamine and tyramine have been considered as the most toxic products in wine, and putrescine and cadaverine could potentiate these effects [4]. The formation of BAs was traditionally controlled by avoiding the growth of spoilage bacteria, decreasing the amino acid precursors and inoculating starter cultures with negative decarboxylase activity [6,7]. Driven by greater awareness of the importance of food quality and safety by consumers, the methods for degradation of BAs in fermented foods have been explored. Biological enzymatic degradation of BAs would be a safe and economic way while avoiding the production difficulties. Two *Lactobacillus plantarum* strains (named NDT 09 and NDT 16) isolated from red wine were able to degrade 22% of tyramine and 31% of putrescine, respectively [14]. Three different strains of *Brevibacterium linens* were utilized to eliminate tyramine and histamine in cheese [6], and the strain *K. varians* LTH 1540, it was also found, could degrade tyramine during sausage ripening [15]. Two lactic acid bacteria were used to degrade 50%–54% of histamine in fish silage [16]. However, the relationship between BAs degradation and microbiological enzymes of the strains has not been explored yet.

In this work, a *Lactobacillus plantarum* was obtained from CRW which could degrade BAs. The optimal industrial conditions of the selected strain were analyzed, and the microbiological amine oxidase enzymes were identified and biochemically characterized. Our results could receive considerable interest by providing a green industrial strategy to control the BAs contents in the rice wine and improve the safety consumption of the fermented foodstuffs.

## 2. Materials and Methods

### 2.1. Materials

Man Rogosa Sharpe agar (MRS) medium was obtained from Oxoid. Ltd. (Basingstoke, Hants, UK). The BA standards were purchased from Sigma-Aldrich (St. Louis, MO, USA). Bacterial genomic DNA extraction kit was obtained from Tiangen (Beijing, China). Ultra-pure water was obtained from a Millipore purification system (>18.3 MΩ·cm). Formic acid, methanol and acetonitrile used in the preparation of the mobile phase were of LC-MS grade. All other chemicals used were of analytical grade.

### 2.2. Strains Screening and Identification

Fermentation broths were collected at the later stage from a typical rice wine production process in Shaoxing (Zhejiang, China). The suspension was filtered through four layers of sterile gauze to remove the unliquefied rice and sealed in a sterile plastic bottle. One gram of fermentation broths was diluted 10-fold by a 0.85% NaCl solution and routinely subcultured 5 to 10 times on MRS medium to obtain purified clones. The screening medium designed was based on the method of Landete [17] to obtain the bacteria that could decrease biogenic amine content. These strains isolated were kept frozen at −20 °C in a sterilized mixture of culture medium and glycerol (50:50, *v*/*v*) according to the methods described by García-Ruiz [18], and further identified by 16S rRNA gene sequencing.

### 2.3. HPLC Determination of Biogenic Amines

Eight biogenic amines of Histamine (HIS), tyramine (TYR), putrescine (PUT), cadaverine (CAD), phenylethylamine (PHE), tryptamine (TRY), spermine (SPM) and spermidine (SPD) were analyzed according to the method of Callejon, Sendra [13] with slight modifications. The individual strains were cultured on MRS, and 10^7^ cfu/mL were inoculated with the MRS liquid medium contaminated with 50 mg/L of each amine at pH 5.5. After 48 h incubation at 30 °C, the reaction was stopped by adding HCl. Samples were centrifuged at 8000 rpm for 15 min and the supernatant was pipetted into a screw-capped vial. The pre-column derivatization procedure using dansyl chloride as derivatization reagent was performed according to the report of Yongmei, Xin [12]. The samples were filtered through 0.22 μm millipore syringe filters and analyzed by RP-HPLC using on LC-20A HPLC system (Shimadzu, Kyoto, Japan) with an Agilent C18 column (250 mm × 4.6 mm, 300 A pores, 5 μm particles, Agilent Technologies, Inc., Santa Clara, CA, USA). The column temperature was kept at 30 °C and the detection wavelength was 254 nm with a flow rate of 1.0 mL/min by using water (A) and methanol (B) as eluents. The gradient elution program consisting of a linear gradient from 65% to 70% B in 7 min followed by from 70% to 80% B in 13 min and 3 min isocratic elution.

The percentage of BAs degradation was calculated based on the HPLC data as following,

BAs degradation (%) = (C_control_ − C_strain_)/C_control_

where C_control_ was the concentration of the BAs in the control medium and C_strain_ was the concentration of the BAs in the medium incubated with the strain.

### 2.4. Bacterial Growth Analysis

The bacterial growth was measured according to the methods described by Cui [19]. Briefly, the isolated lactic acid bacteria (LAB) strains were diluted to 10^5^ cfu/mL in MRS liquid medium, and the pH and optical density (OD_600 nm_) of medium was checked at 28 °C, 33 °C and 37 °C for 36 h, respectively.

### 2.5. The Bacterial Starter Application in Pilot Scale Fermentation

**Figure 1:**
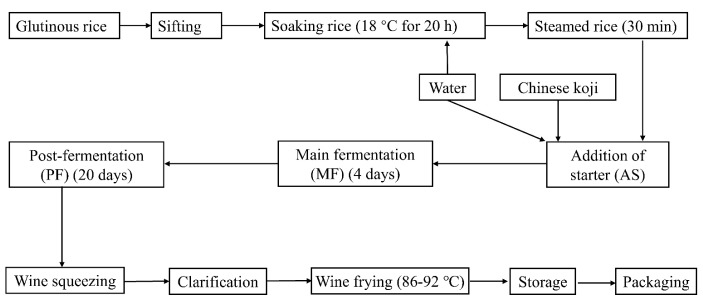
Diagram of the Chinese rice wine production process.

A pilot fermentation was performed according to the methods described by Zhang, Xue [10] with modifications (Figure 1). Glutinous rice (12 kg) was soaked at 18 °C for 20 h and steamed for 30 min. After naturally cooling to room temperature (about 25 °C), the steamed rice was transferred into a 33 L wide-mouth bottle to which 14.5 kg water, 1.5 kg Chinese koji (unique saccharifying agent including molds, yeasts and bacteria, obtained from COFCO Shaoxin wine Co., Ltd., Shaoxin, China) were added. The main fermentation was carried out at 33 °C for 4 days with intermittent oxygen filling, and post-fermentation was then carried out at 28 °C for 20 days. The isolated strain with 10^5^ (low level), 10^6^ (middle level) and 10^7^ (high level) cfu/mL was added into the CRW at the main fermentation and post-fermentation stage, respectively. After filter pressing, clarification, wine frying and sterilization (90 °C for 3 min), finished Chinese rice wines were obtained. Ten milliliters of fermentation broths were taken from different fermentation stages, including addition of starter (AS); main fermentation (MF); post-fermentation 5d (PF5d); post-fermentation 10d (PF10d); and post-fermentation 20d (PF20d)), to analysis the changes in the BAs contents by using the HPLC method. According to the previous studies [20,21], pH, alcohol content, total sugar, total acid, non-sugar solid and amino acid nitrogen of CRW were analyzed by using official methods (Chinese National Standard GB/T 13662-2008). Sensory evaluation of CRW was conducted by 30 panelists (15 males and 15 females) who have professional training certificates. The procedure was conducted in a sensory laboratory following GB/T 13662-2008 and ISO 4121. A total of 11 sensory attributes of appearance (color and turbidity), aroma (alcohol, fruit and cereal), taste (sweet, sour and bitter), mouthfeel (astringency, continuation and full body) and harmony were chosen to characterize the sensory properties using quantitative descriptive analysis involving a 0–9 ten-point linear scale (0: none; 1–2: very weak; 3–4: ordinary; 5–6: moderate; 7–8: strong; 9: very strong).

### 2.6. Separation of the Amine Oxidases

Cell-free extracts were obtained by using the method of Callejon [22]. The bacterial cells from a 1 L culture were collected by centrifugation at 10,000 rpm, 20 min at 4 °C and washed twice with 50 mM sodium phosphate buffer (PBS), pH 7.4. The samples were resuspended in PBS buffer containing 1 mM of phenyl methylsulfonyl fluoride (PMSF) as protease inhibitor. Cell-free extracts were obtained by disrupting the bacterial cells with 1 g of 106 μM diameter glass beads in a Mikro-dismenbrator^®^ Sartorius: 10 cycles of 40 s, alternating 5 cycles of disruption with a cooling step of 5 min in ice. The samples were centrifuged at 13,000 rpm for 15 min (PrismR, Labnet, USA), and supernatants were saved at −20 °C until use. The protein content was determined by using the bicinchoninic acid assay kit (BCA, Solarbio, Beijing, China). Monoamine oxidase (MAO) assay kit and diamine oxidase (DAO) assay kit (Jiancheng Institute, Nanjing, China) were both used to determine the amine oxidase activity. The MAO assay kit was based on the ability of MAO to form H_2_O_2_ substrate, which could be determined by a fluorimetric method. The DAO assay kit was based on the oxidation of PUT to pyrroline plus NH_3_ and H_2_O_2_, which can be determined by the fluorimetric method.

The cell-free extracts were further ultracentrifuged at 47,000 rpm for 1 h, and the supernatant was precipitated by 75% saturation of ammonium sulfate precipitation [22]. The protein was redissolved with 50 mM PBS and were loaded onto a Sephadex G-100 column (1.6 cm × 70 cm) followed by a linear gradient elution with a flow rate of 1 mL/min. The protein fraction was collected and measured at 280 nm by using a HD-93-1 spectrophotometer (Purkinje General Instrument Co. Ltd., Beijing, China). There fractions were collected (P1, P2 and P3, Supplement Figure S1), and were then concentrated and freeze-dried. The degradation ability of the fractions was further evaluated by incubation with 50 mg/L eight biogenic amines at pH 4.0, 33 °C for 2 h.

### 2.7. Identification of the Amine Oxidases

The fractions separated from the cell-free extracts were digested with trypsin (Promega, Madison, WI, USA) overnight at 37 °C and were identified by LC-MS/MS using the Easy nLC-1000 nano ultra-high-pressure system (Thermo Fisher Scientific, San Jose, CA, USA) coupling with a Q Exactive mass spectrometer (Thermo Fisher Scientific, San Jose, CA, USA). The peptide mixture was loaded onto a Zorbax 300SB-C18 peptide traps (Agilent Technologies, Wilmington, DE, USA) in buffer A (0.1% Formic acid) and separated with a linear gradient of 4%–50% buffer B (80% acetonitrile and 0.1% formic acid) for 50 min, 50%–100% B for 4 min, and held at 100% B for 6 min at a flow rate of 250 nL/min. The mass spectrometer was operated in positive ion mode. MS data was acquired using a data-dependent top10 method dynamically choosing the most abundant precursor ions from the survey scan for high-energy collisional dissociation (HCD) fragmentation and was searched by using MASCOT engine and Proteome Discoverer 1.3 against the local uniport_lactobocilluspiantarum database.

### 2.8. Enzymatic Properties of the Amine Oxidases

Effects of temperatures (15, 20, 25, 28, 30, 35, 40, 80 °C at pH 4.0 for 2 h), pH (3.0–5.0) at 30 °C for 2 h, and metal ions (0.2 mol/L, copper ion, ferrous ion, zinc ion, calcium ion and magnesium ion) at 30 °C for 2 h (pH 4.0) on the amine oxidase degradation activity were further investigated.

### 2.9. Statistical Analysis

All samples were prepared in three independent and each was analyzed in triplicate by the analysis of variance (ANOVA). The results were considered significant at *p* ≤ 0.05 by the Duncan test.

## 3. Result

### 3.1. Strains Screening and Identification

A total of 61 strains were isolated from the five major stages (soaking rice, steamed rice, addition of starter, main fermentation and post-fermentation, Figure 1) of CRW fermentation. After screening their potentials to degrade/eliminate the contents of BAs, about 30% of strains were able to degrade BAs even though most of them degraded BAs to less than 10% extents (results not known). Only one strain drew attentions for more than 40% degradation efficiency of the BAs (Table 1). 16S rDNA sequencing identified that the strain had 100% similarity in 16S rDNA sequences to *Lactobacillus plantarum* CAU 3823 (GenBank accession no. MF424991.1). In the details, *Lactobacillus plantarum* CAU 3823 was a *L. plantarum* that exhibited the greatest potential for BAs degradation, as 56% degradation, for TRY, 41% for PHE, 42% for PUT, 43% for CAD, 40% for TYR, 45% for HIS, 44% for SPD and 43% for SPM, which should be considered in the further analysis.

### 3.2. The Bacterial Growth Ability

**Figure 2:**
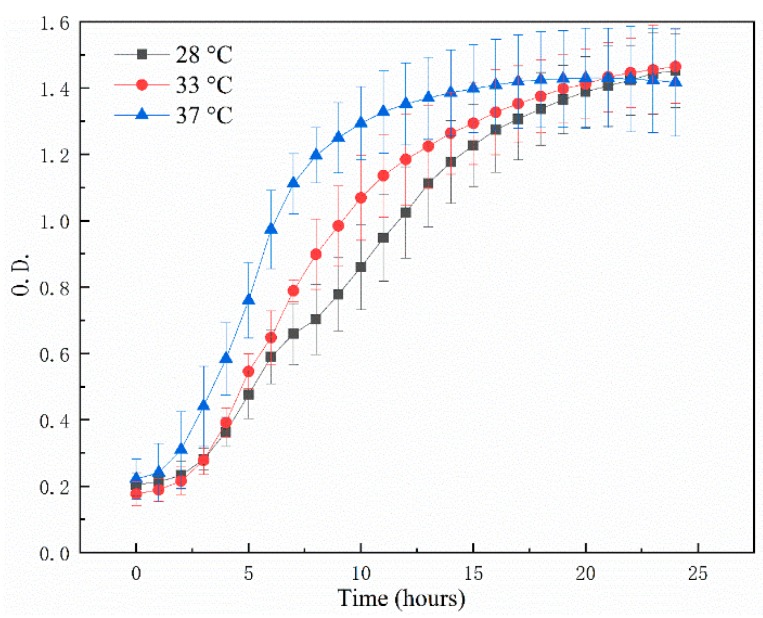
The growth ability of *L. plantarum* CAU 3823 at different temperatures (28 °C, 33 °C and 37 °C). Growth curves are representative of all determinations.

The growth ability of *L. plantarum* CAU 3823 at different temperatures (28 °C, 33 °C and 37 °C) was shown in Figure 2. *L. plantarum* CAU 3823 was able to grow at different temperatures, showing OD_600_ > 1 at main fermentation temperature (33 °C) for 9–25 h and post-fermentation temperature (28 °C) for 12~25 h. The maximum OD_600_ value of 1.4 was found at different temperatures at 25 h of growth, suggesting the good growth trends indicated that *L. plantarum* CAU 3823 could be used in industry producing CRW fermentation.

### 3.3. Changes in the BAs Induced by L. plantarum in Pilot Scale Fermentation

**Figure 3:**
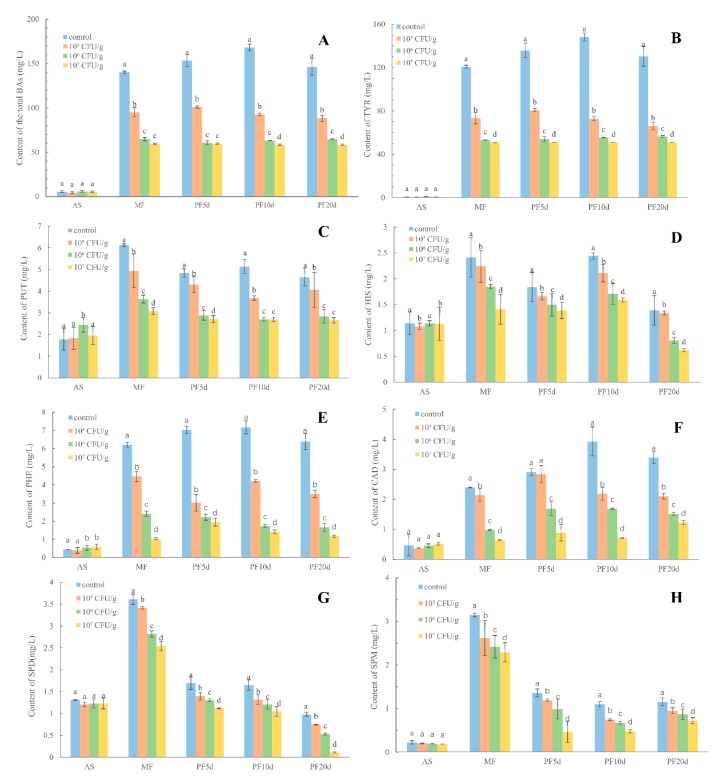
The contents of the total biogenic amines (BAs, **A**), tyramine (TYR, **B**), putrescine (PUT, **C**), Histamine (HIS, **D**) phenylethylamine (PHE, **E**), cadaverine (CAD, **F**), spermidine (SPD, **G**) and spermine (SPM, **H**) in Chinese rice wine adding different level of *L. plantarum* CAU 3823 at the post-fermentation and main fermentation stage during different fermentation stages (addition of starter (AS); main fermentation (MF); post-fermentation 5d (PF5d); post-fermentation 10d (PF10d); post-fermentation 20d (PF20d)).

To investigate the capability to degrade BAs of *L. plantarum* CAU 3823 to the BAs in pilot scale fermentation of CRW, RP-HPLC was applied to quantify the contents of BAs in CRW incubation with various levels (10^5^, 10^6^ and 10^7^ cfu/mL) of *L. plantarum* CAU 3823 as extra starter during fermentation, and the results are shown in Figure 3. Compared to control group, the total contents of BAs in CRW with *L. plantarum* CAU 3823 were significantly lower (*p* < 0.05) during the entire fermentation period (Figure 3A). The degradation percentages of BAs were 32%, 54% and 58%, respectively, at low, middle and high level of *L. plantarum* CAU 3823 at the main fermentation stage, suggesting the dose dependent manner. Total content of BAs was significantly reduced to 34%, 60% and 61% at low, middle and high levels of *L. plantarum* CAU 3823, respectively, at 5th day of post-fermentation, and similar degradation efficiency was obtained in the 10th day of post-fermentation and 20th day of post-fermentation, respectively.

The degrading abilities of *L. plantarum* CAU 3823 to TRY, PUT, HIS, CAD, PHE, SPD and SPM were also studied in Figure 3B–H, respectively. A marked decrease in the contents of BAs was observed during fermentation with the increasing of the strain content. As the most content of BAs detected in Chinese rice wine, TRY was degraded by *L. plantarum* CAU 3823 with the degradation rate of 39% at low level, 56% at middle level and 58% at high level strain at main fermentation; 41% at low level, 60% at middle level and 62% at high level strain at post-fermentation 5d; 51% at low level, 63% at middle level and 66% at high level strain at post-fermentation 10d; and 49% at low level, 57% at middle level and 61% at high level strain at post-fermentation 20d (Figure 3B). Similar degradation efficiency to PUT, HIS, CAD, PHE, SPD and SPM was also found as follows: PUT with 13% reduction at low level, 39% at middle level and 43% at high level strain; HIS with 4% at low level, 42% at middle level and 55% at high level; PHE with 45% at low level, 74% at middle level and 82% at high level; CAD with 38% at low level, 55% at middle level and 55% at high level; SPD with 23% at low level, 46% at middle level and 89% at high level; SPM with 25% at low level, 50% at middle level and 75%, respectively, at high level at the end of post-fermentation. Overall, more than 40% contents of BAs could be degraded incubation with *L. plantarum* CAU 3823 at the middle and high levels than the one at low level during fermentation.

### 3.4. Total Acid and pH in Pilot Scale Fermentation

As shown in Table 2, the changes in total acid and pH value of Chinese rice wine when different levels of *L. plantarum* CAU 3823 were added during fermentation were investigated, to evaluate the effect of this strain on the quality of CRW. At the initial stage of starter addition, there was no difference (*P* > 0.05) in lactic acid content and pH value among the four CRW samples. The total acid content of the CRW showed a slightly increase from 6.53 at low level to 6.86 g/L at middle level strain incubated with *L. plantarum* CAU 3823 at the end of post-fermentation, compared to the control group of 5.94 g/L. However, the total acid of CRW of 9.14 g/L incubated with high level of *L. plantarum* CAU 3823 indicated the over-acidification.

### 3.5. Alcohol Content, Total Sugar, Non-Sugar Solid and Amino Acid Nitrogen in Pilot Scale Fermentation

The effects of *L. plantarum* CAU 3823 on the alcohol content, total sugar, non-sugar solid and amino acid nitrogen in the Chinese rice wine were analyzed after production process. As presented in Table 3, there was no notable change in alcohol, amino acid nitrogen and total sugar contents among the CRWs incubated with low and middle level of *L. plantarum* CAU 3823. The non-sugar solid was markedly higher (*p* < 0.05) when CRW was fermented involving with the selected strain.

### 3.6. Sensory Evaluation

**Figure 4:**
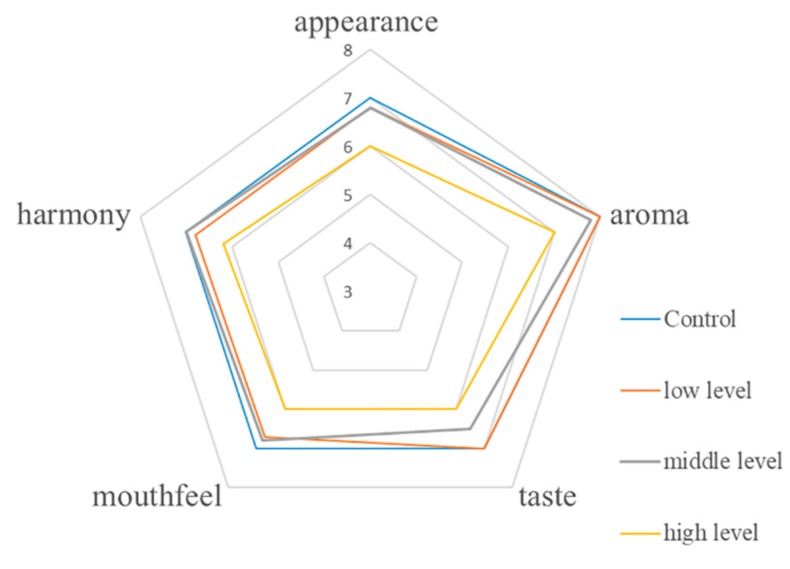
Average radar map of the Chinese rice wine including different level of biogenic amine-reduced *Lactobacillus plantarum* based on sensory scores.

The sensory characteristics of CRW adding with different levels of the isolated strain were described by the 30 sensory panelists. As presented in Figure 4, CRW with high level of strain exhibited the lowest score (appearance 6, aroma 7, taste 6, mouthfeel 6 and harmony 6.2) among the four CRW samples. No significant difference was observed between the CRW incubated with middle level and low level strain compared to the control CRW (*p* > 0.05), indicating *L. plantarum* CAU 3823 with low and middle level would not have an influence on the sensory behaviors of the Chinese rice wine.

### 3.7. Purification and Identification of the Amine Oxidases

To gain a deeper insight into the amine-degrading activity exhibited by *L. plantarum* CAU 3823, LC-MS/MS experiments were designed to show whether the amine oxidases existed in the strain. Cell-free extracts were obtained at a protein concentration of 5.5 mg/mL (Table 4). The MAO activity was 36.9 U/mg and the DAO activity was 128 U/L at 37 °C, pH = 7 in the cell-free extracts **(**Table 4**)**. Three fractions were collected from a Sephadex G-100 column (Supplement Figure S1), and little DAO activity was detected in all three fractions, but only fraction 1 showed a good MAO activity of 19.8 U/mg compared to fraction 2 of 2.4 U/mg, and no amine oxidase activity was determined in fraction 3, which might be due to the low protein concentration.

To further investigate the amine degradation ability, the BA degradation rate (%) was calculated by incubating the three fractions with the eight BAs at pH 4.0, 33 °C for 2 h (Table 5). The BAs contents in fraction 1 significantly declined with the degradation rate of 41.9% for TYR, 41.1% for HIS, 40.3% for PUT, 44.3% for PHE, 41.1% for CAD, 41% for SPD, 43.5% for SPM and 47.9% for TRY. However, there were slight or little changes observed in the BA contents in the Fractions 2 and 3.

The fraction 1 was further identified by using LC-MSMS. Ten proteins including 9 amine oxidase proteins were identified in fraction 1, and hereinto, 8 amine oxidase proteins were monoamine oxidases, including 4 amine oxidase [flavin-containing] A (accession: P58027, P21396, Q5NU32 and A0A011QTL0), 2 amine oxidase [flavin-containing] B (accession: Q5RE98 and A0QU10), 1 monoamine oxidase [flavin-containing] (accession: A0A375EQX7) and 1 monoamine oxidase (accession: U2EF11) (Supplement Table S1). The MWs of the amine oxidases were closer and range from 46 to 60 kDa.

### 3.8. Amine Oxidases Assays

**Figure 5:**
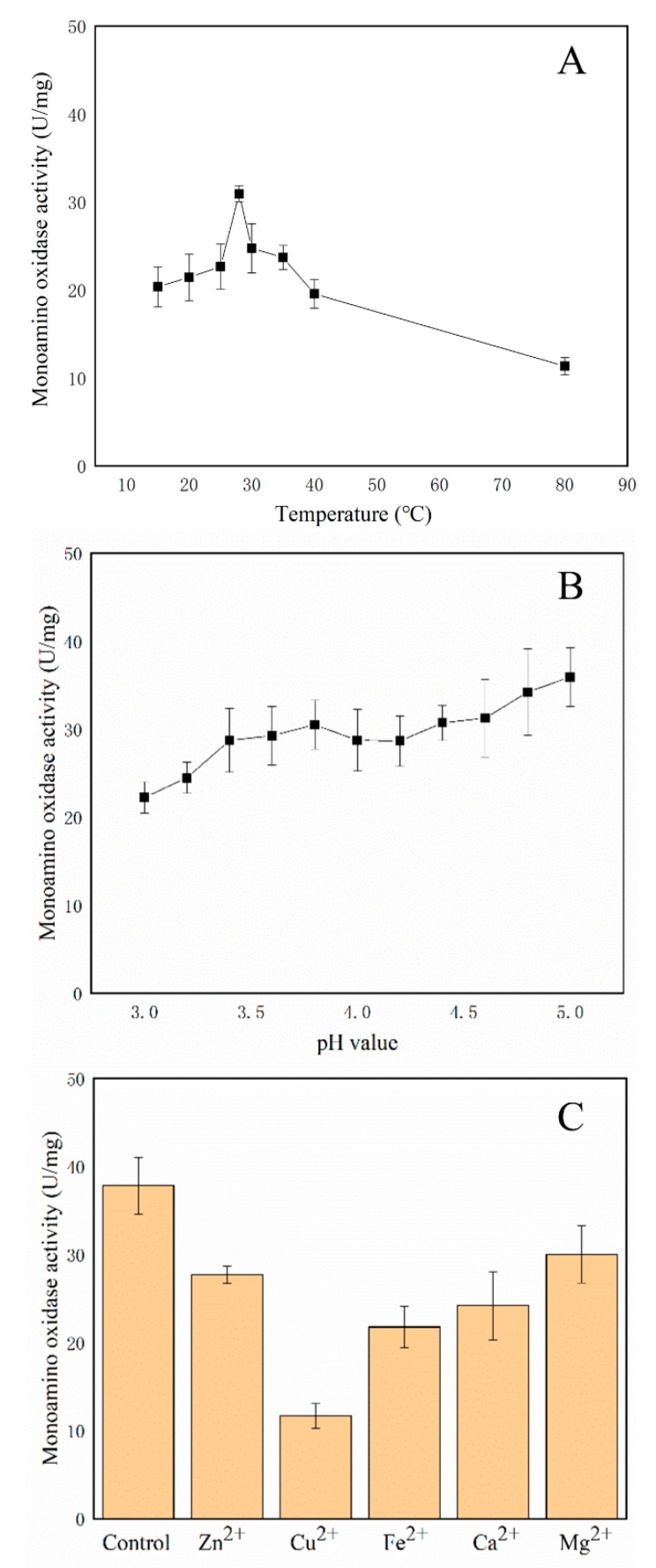
The monoamine oxidase (MAO) activity in the amine oxidase mixture at different temperatures (**A**) at pH 4.0 for 2 h; different pH (**B**) at 30 °C for 2 h and different metal ions (**C**) at 30 °C for 2 h (pH 4.0).

As shown in Figure 5, the purified amine oxidases mixture (fraction 1) retained its activity in a wide temperature range from 15 to 80 °C and was shown to maintain the 50% MAO activity after on heat treatment at 80 °C for 2 h. The optimal temperature for the amine oxidase activity was 28 °C and the MAO activity was 36.9 U/mg (Figure 5A). The MAO activities increased from 22.3 U/mg to 35.9 U/mg accompanied by the pH value from 3.0 to 5.0 while the amine oxidases were incubated at 30 °C for 2 h (Figure 5B). All the ions could inhibit the MAO activity, as 73%, 31%, 58%, 64% and 79% activity retained when adding 0.2 mol/L Zn^2+^, Cu^2+^, Fe^2+^, Ca^2+^ and Mg^2+^, respectively (Figure 5C).

## 4. Discussion

Biogenic amines are considered as potential health risks since high amounts of them can lead to a series of health problems. The intake of foods with high level of BAs could induce the release of adrenaline and noradrenaline, provoking gastric acid secretion, increased cardiac output, migraine, tachycardia, increased blood sugar levels, and higher blood pressure [23]. Several researches supported the view that the BAs were formed in winemaking mainly by lactic acid bacteria carrying specific metabolic pathways that convert precursor amino acids into BAs [24]. In contrast, there is a lack of studies concerning BAs degradation by food sourced micro-organisms in wine, especially in Chinese rice wine.

In this paper, a *L. plantarum* CAU 3823, isolated from Chinese rice wine, can degrade more than 40% of the BAs, especially the five major BAs of TYR, PUT, HIS, PHE and CAD in Chinese rice wine. A similar research in grade wine showed that only one strain, *L. casei* IFI-CA 52, showed a strong ability to degrade the BAs (54% HIS, 55% TRY and 65% PUT) isolated from wine/ grape cell cultures of 85 strains [18]. However, the histamine-degrading ability of *L. casei* IFI-CA 52 was only 17% when addition of 12% ethanol, suggesting that the ability of *L. casei* IFI-CA 52 to reduce amine concentrations in wines would be rare. Regrettably, the ability of this strain to degrade other BAs was not analyzed. Moreover, a pilot scale fermentation, rather than addition of ethanol, would be a better choice to simulate accurately the complicated wine matrix.

In our experiment, pilot scale fermentation tests had proved that *L. plantarum* CAU 3823 was competent to be used as an extra starter in CRW industrial producing. Chinese koji was added at the beginning of brewing, which could bring in lots of bacteria, thus the BAs accumulated significantly at the beginning [13]. The BAs concentration showed a notably increase in the common CRW (the control group) from the starter addition stage to 10-days post-fermentation, indicating the proliferation of bacteria [13]. The concentration of BAs decreased at the end of post-fermentation, which might be due to the bacteria growth inhibition as the total acid increased during fermentation. According to our results, *L. plantarum* CAU 3823 could degrade the BAs in the CRW brewing process, and the formation of biogenic amines was further degraded by increasing the dose of strain. In this study, HIS, TYR, PUT and CAD were degraded significantly during the pilot scale fermentation, especially TYR, which indicated *L. plantarum* CAU 3823 could provide a more safety traditional fermented beverage for consumers.

Identification of functional microorganisms in CRW to reduce the formation of BAs has received more interest. Liu, Yu [13] utilized an in vivo screening process based on the next-generation sequencing technology to find BA-decreasing microorganism in CRW, and three *Lactobacillus* strains were detected that would not form biogenic amines, but only *L. plantarum* JN01 could grow under 15% ethanol, and the wine could form an unpleasant rancidity taste and more than 8 g/L total acid when the *L. plantarum* JN01 was more than 0.01 gDCW/t. Indeed, high level of functional bacteria could bring about unsatisfactory changes in CRW. A similar trend found in the current study showed that the total acid increased, and alcohol content decreased when 10^7^ cfu/mL (high level) of *L. plantarum* CAU 3823 was added into the fermentation mash. Although the sensory scores were also decreased, the whole CRW was within the acceptable range for consumers at high level of the strain. Therefore, *L. plantarum* CAU 3823 could be the best choice to date to decrease BAs in CRW.

As a traditional alcoholic beverage, total sugar, alcoholic degree, pH value, total acid, amino acid nitrogen and non-sugar solid would play important roles in the flavor, taste and nutrition of Chinese rice wine [22]. Although high level (10^7^ cfu/mL) of *L. plantarum* CAU 3823 could degrade the BAs maximally, undesirable influence on the acceptability was also noteworthy. Low level (10^5^ cfu/mL) and middle level (10^6^ cfu/mL) of *L. plantarum* CAU 3823 could eliminate the negative effect on the qualities of the wine, and what’s more important, similar sensory characteristics were obtained in CRW. Thus, to degrade the content of BAs in CRW to the highest extent, middle level (10^6^ cfu/mL) of the *L. plantarum* could be chosen in the CRW fermentation process.

Non-sugar solids, a major nutrition indicator to evaluate the quality grade of CRW, are mainly composed of dextrin, glycerin, non-volatile acid, protein and hydrolysates [25]. Interestingly, the content of non-sugar solids was increased remarkably when *L. plantarum* CAU 3823 was used, especially at middle level (10^6^ cfu/mL), which provided a novel insight that the *L. plantarum* CAU 3823 could produce more non-sugar solids in CRW and thus have potential nutritional values.

BA can be converted into products via oxidation by microorganisms which can be used as a carbon and/or energy source or as a nitrogen source [26]. Limited studies attributed these transformations to amine oxidase activity derived from microorganisms. Yagodina [27] reported that flavoprotein oxidases existing in some microorganisms could catalyze the oxidation of BAs. Sekiguchi [28] found a histamine oxidase in the actinobacteria *Arthrobacter crystallopoietes* KAIT-B-007 isolated from soil. In this study, the amine oxidases from *L. plantarum* CAU 3823 were purified and characterized. Nine amine oxidase proteins, a mixture from *L. plantarum* CAU 3823, contributed the most of amine-degrading ability of *L. plantarum* CAU 3823. Eight MAOs were identified and thus confirmed a good monoamine oxidase activity shown in fraction 1. Amine oxidases can be divided into two subfamilies based on the cofactor they contain. MAO (EC 1.4.3.4) are a family of enzymes containing flavin that catalyze the oxidation of monoamines, employing oxygen to clip off their amine group [29]. The amine oxidases containing copper as cofactor (EC 1.4.3.6) are homodimers, which contain three subclass, namely, diamine oxidase, primary-amine oxidase and diamine oxidase [30]. Amine oxidase [flavin-containing] A and B can catalyze the oxidative deamination of biogenic amines [31]. Amine oxidase [flavin-containing] B that in humans was encoded by the MAOB gene could preferentially degrade PHE [32], which confirmed 44.3% PHE degradation in fraction 1. An “aromatic cage” has been found to play a steric role in substrate binding and in flavin accessibility and helps to increase the substrate amine nucleophilicity [33], which might enhance BA degradation. It is noted that no diamine oxidase was identified although cell-free extracts showed diamine oxidase activity.

To provide a seemingly feasible solution to degrade the BAs in foodstuffs, the biochemical character assays of the amine oxidases mixture from *L. plantarum* CAU 3823 were designed. The enzymes were very thermostable, as the activity remained stable at 80 °C, and were fully stable over the pH range of 3–5. Similar results were reported that a putrescine oxidase from *Rhodococcus erythropolis* NCIMB 11540 could be stable at 50 °C for 2 h [34] and a thermostable histamine oxidase was found in *Arthrobacter crystallopoietes* KAIT-B-007 [29]. These results indicated that the amine oxidases could be stable to use in fermented food processing.

## 5. Conclusions

In this paper, *Lactobacillus plantarum* CAU 3823 was a *L. plantarum* originating from Chinese rice wine which could effectively degrade the BAs. Middle level (10^6^ cfu/mL) of *L. plantarum* could be an optimal choice to decrease the BAs maximally while keeping the CRW character and quality in the pilot scale fermentation. Nine amine oxidase proteins were identified from *L. plantarum* using Sephadex separation followed by LC-MS/MS analysis. The enzymes were very thermostable and fully stable at pH 3–5. All the ions can inhibit the amine oxidase to an extent. *L. plantarum* seemed to be an interesting species displaying BAS degradation, both in culture media conditions and in CRW fermentation, suggesting its suitability as a commercial malolactic starter. This paper provided an efficient method to decrease the biogenic amine contents in the traditional fermented food made by multiple microbes like wine, rice wine, sausages, vinegar, cheese, kimchi and so on.

## Figures and Tables

**Table 1:** Percentage (%) of degradation of the biogenic amines by *Lactobacillus plantarum* CAU 3823 from Chinese rice wine ^a^.

Strains	Tryptamine	Phenylethylamine	Putrescine	Cadaverine	Tyramine	HISTAMINE	Spermidine	Spermine
*Lactobacillus plantarum* CAU 3823	55.95 ± 6.59	40.85 ± 9.87	41.82 ± 7.97	42.79 ± 7.76	40.12 ± 8.09	44.72 ± 7.56	43.51 ± 8.39	42.56 ± 8.41

^a^ 10^7^ cfu/mL of *Lactobacillus plantarum* CAU 3823 was incubated in the Man Rogosa Sharpe agar (MRS) liquid medium contaminated with 50 mg/L of each amine at pH 5.5 for 48 h.

**Table 2:** Changes in the total acid and pH in the Chinese rice wine adding different level of *L. plantarum* CAU 3823 at the post-fermentation and main fermentation stage during different fermentation stages (addition of starter; main fermentation; post-fermentation 5d; post-fermentation 10d; post-fermentation 20d).

	Addition the Selected Strain (cfu/mL)	The Addition of Starter	Main Fermentation	Post-Fermentation 5d	Post-Fermentation 10d	Post-Fermentation 20d
Total acid (g/L)	Control	6.03 ± 0.22 ^a^	3.81 ± 0.15 ^a^	4.91 ± 0.07 ^a^	5.55 ± 0.33 ^a^	5.94 ± 0.20 ^a^
	10^5^ (low level)	6.17 ± 0.13 ^a^	4.93 ± 0.13 ^b^	5.41 ± 0.19 ^b^	5.92 ± 0.19 ^a^	6.53 ± 0.13 ^b^
	10^6^ (middle level)	5.92 ± 0.19 ^a^	6.01 ± 0.14 ^c^	6.51 ± 0.14 ^c^	6.74 ± 0.44 ^b^	6.86 ± 0.13 ^d^
	10^7^ (high level)	6.03 ± 0.15 ^a^	6.04 ± 0.10 ^c^	7.06 ± 0.09 ^c^	8.01 ± 0.23 ^c^	9.14 ± 0.45 ^c^
pH	Control	6.33 ± 0.19 ^a^	4.04 ± 0.12 ^a^	4.19 ± 0.05 ^a^	4.21 ± 0.03 ^b^	4.14 ± 0.12 ^a^
	10^5^ (low level)	6.37 ± 0.28 ^a^	4.00 ± 0.14 ^a^	4.36 ± 0.12 ^a^	4.12 ± 0.07 ^a^	3.99 ± 0.16 ^a^
	10^6^ (middle level)	6.45 ± 0.22 ^a^	3.84 ± 0.16 ^a^	4.34 ± 0.08 ^a^	4.45 ± 0.13 ^b^	3.87 ± 0.12 ^a^
	10^7^ (high level)	6.43 ± 0.23 ^a^	3.71 ± 0.04 ^b^	4.24 ± 0.12 ^a^	4.32 ± 0.12 ^b^	3.63 ± 0.03 ^b^

Presented data (mean ± standard deviation) are the mean values of three independent samples and each analyzed in triplicate. Values in a column with different superscripts differ significantly (*p* < 0.05).

**Table 3:** The alcohol content, amino acid nitrogen, total sugar and non-sugar solid in the Chinese rice wine after production process.

Addition the Selected Strain (cfu/mL)	Alcohol Content (% vol)	Amino Acid Nitrogen (g/L)	Total Sugar (g/L)	Non-Sugar Solid (g/L)
Control	11.52 ± 0.23 ^a^	1.44 ± 0.11 ^a^	31.98 ± 1.37 ^a^	39.81 ± 0.33 ^a^
10^5^ (low level)	11.49 ± 0.35 ^a^	1.28 ± 0.35 ^a^	15.35 ± 2.34 ^b^	62.34 ± 0.32 ^c^
10^6^ (middle level)	10.33 ± 0.41 ^b^	0.82 ± 0.13 ^b^	11.98 ± 3.25 ^b^	71.52 ± 0.18 ^d^
10^7^ (high level)	9.29 ± 0.25 ^c^	0.59 ± 0.02 ^c^	10.97 ± 2.23 ^b^	51.16 ± 0.25 ^b^

Presented data (mean ± standard deviation) are the mean values of three independent samples and each analyzed in triplicate. Values in a column with different superscripts differ significantly (*p* < 0.05).

**Table 4:** The protein concentration, monoamine oxidase activity and diamine oxidase activity of the cell-free extracts (37 °C, pH = 7).

	Protein Concentration (mg/mL)	Monoamine Oxidase Activity (U/mg)	Diamine Oxidase Activity (×10^−4^ U/mg)
Cell-free extracts	5.5	36.9 ± 5.2	1.3 ± 0.1
Fraction 1	3.1	19.8 ± 2.6	ND
Fraction 2	1.6	2.4 ± 1.2	ND
Fraction 3	0.5	ND	ND

ND = Not determined.

**Table 5:** Degradation percentages (%) of the eight biogenic amines in the three fractions by Sephadex separation incubation with 50 mg/L of the eight biogenic amines at pH 4.0, 33 °C for 2 h.

	Tryptamine	Phenylethylamine	Putrescine	Cadaverine	Histamine	Tyramine	Spermidine	Spermine
Fraction 1	47.9	44.3	40.3	41.1	41.1	41.9	41	43.5
Fraction 2	ND	0.3	0.7	ND	1.2	3.8	ND	ND
Fraction 3	ND	ND	ND	ND	ND	ND	ND	ND

ND = Not determined.

## Supplementary Materials

The following are available online at https://www.mdpi.com/2304-8158/8/8/312/s1. Figure S1: The three fractions separated by a Sephadex G-100 column, Table S1: A group of proteins by Sephadex separation followed by LC-MS/MS analysis in fraction 1.
