# Supplementary material for: Identification of a Lactic Acid Bacteria to Degrade Biogenic Amines in Chinese Rice Wine and Its Enzymatic Mechanism
Source: Foods. 2019 Aug 2;8(8):312. doi: 10.3390/foods8080312 (PMC6724028; doi:10.3390/foods8080312)
Supplement: Supplementary file 1 [file foods-08-00312-s001.pdf]

Supplement Figure S1. The three protein fractions separated by a Sephadex G-100 column

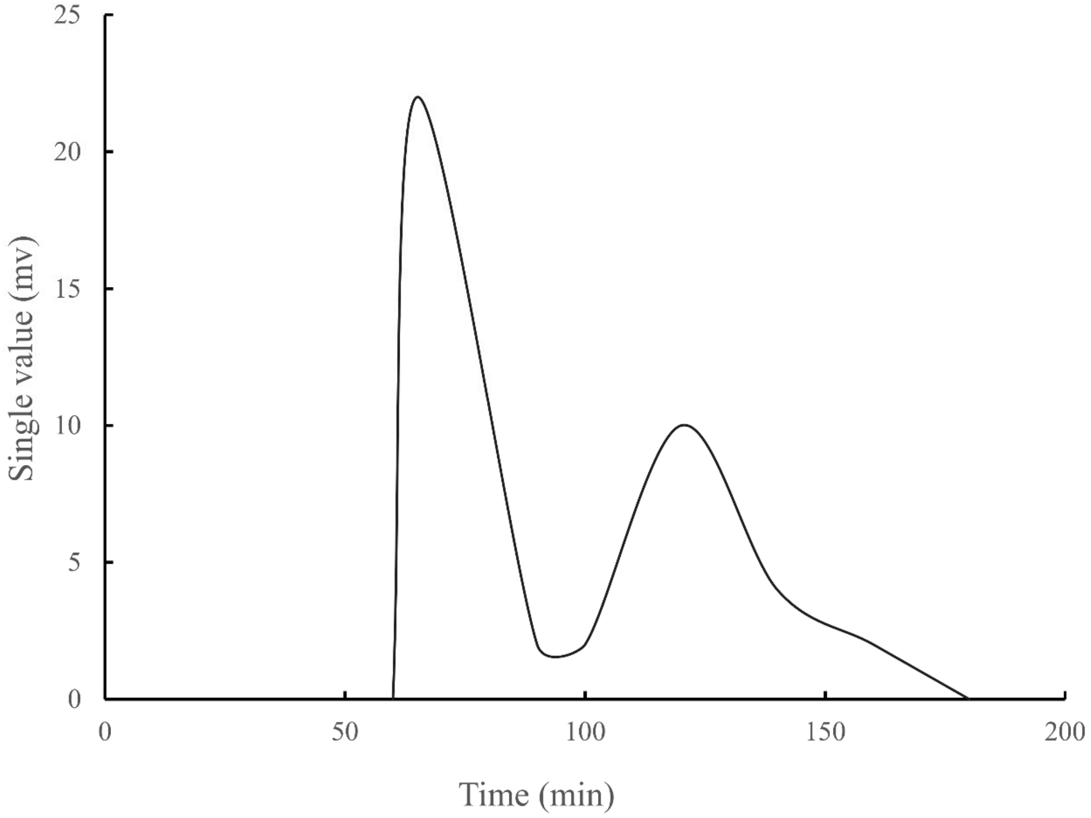


Supplement Table S1: A group of proteins by Sephadex separation followed by LC-MS/MS analysis in fraction 1

| Number | Accession | Molecular weight | Cover % | Description |
|---|---|---|---|---|
| 1 | A0A086LU36 | 195862.76 | 5.15 | Histone lysine-specific demethylase LSD1/BHC110/KDMA1A |
| 2 | P58027 | 60087.46 | 11.39 | Amine oxidase [flavin-containing] A |
| 3 | P21396 | 59507.15 | 10.27 | Amine oxidase [flavin-containing] A |
| 4 | Q5NU32 | 59578.96 | 7.02 | Amine oxidase [flavin-containing] A |
| 5 | Q5RE98 | 58732.15 | 11.35 | Amine oxidase [flavin-containing] B |
| 6 | A0A375EQX7 | 55603.44 | 11.32 | Monoamine oxidase [flavin-containing] |
| 7 | Q39NU1 | 52569.02 | 11.38 | Amine oxidase |
| 8 | A0QU10 | 49303.09 | 15.16 | Amine oxidase [flavin-containing] B |
| 9 | U2EF11 | 47084.7 | 10.34 | Monoamine oxidase |
| 10 | A0A011QTL0 | 46490.35 | 11.94 | Amine oxidase [flavin-containing] A |
